# Biotin-transfer from a trifunctional crosslinker for identification of cell surface receptors of soluble protein ligands

**DOI:** 10.1038/srep46574

**Published:** 2017-04-19

**Authors:** Tammy-Lynn Tremblay, Jennifer J. Hill

**Affiliations:** 1 Human Health Therapeutics, National Research Council Canada, 100 Sussex Dr., Ottawa, ON K1A 0R6, Canada

## Abstract

Here we describe a novel crosslinker and its application as a biotin-transfer reagent to identify cell surface receptors of soluble protein ligands on live cells. This crosslinker contains three functional groups: an aldehyde-reactive aminooxy group, a sulfhydryl, and a biotin (ASB). It is readily synthesized via a 3-step addition reaction using standard solid-phase peptide synthesis methods and commercially available intermediates, allowing access to laboratories without specialized synthetic chemistry capabilities. For the biotin-transfer method, ASB is linked to a protein ligand through the sulfhydryl group in a two-step process that allows the introduction of a disulfide bond between the ligand and the crosslinker. Incubation of the labelled ligand with oxidized live cells leads to the formation of crosslinks with aldehyde-containing glycans on the cell surface receptor. Subsequent reduction of the disulfide bond results in biotin transfer from the ligand to the cell surface receptor. Protein biotinylation that is mediated by ligand binding to its receptor is differentiated from background biotinylation events by comparison with a similarly labelled control protein using comparative proteomic mass spectrometry to quantify streptavidin-bound proteins. Using this method, we successfully identified the cell surface receptors of a peptide hormone, a monoclonal antibody, and a single-domain antibody-Fc fusion construct.

Soluble protein ligands exert their effects on cells through cell surface receptors which initiate cell signaling events. In many cases, the cell surface receptors bound by soluble protein ligands are not known and the identification of these receptors is complicated by their hydrophobic membrane-bound nature. In addition, the growth of synthetic antibody libraries and cell panning approaches has led to a large number of cell-binding antibodies without known antigens leading to an increased need for antigen identification methods for these cell binding ligands^1^.

Frei *et al*. have recently shown that ligand-directed crosslinking using their TRICEPS crosslinker is an effective method for the identification of cell surface binding partners for soluble protein ligands^2^,^3^. In the TRICEPS method, soluble ligands are labelled with the trifunctional TRICEPS crosslinker through an amine reactive group and crosslinks are formed following binding of the labelled ligand to oxidized cells or tissues via hydrazone bond formation between a protected hydrazide group on the TRICEPS crosslinker and aldehyde-containing glycans on the cell surface receptor^4^,^5^. Following trypsin digestion, streptavidin is used to purify crosslinked peptides via the biotin moiety contained in the TRICEPS crosslinker. Peptides belonging to the cell surface receptors are specifically released from the strepatavidin beads by the enzyme N-Glycosidase F (PNGaseF) which cleaves the bond between the glycan and N-linked glycosylated peptides. To differentiate between non-specific crosslinking events and crosslinking events mediated through binding of the ligand to its cell surface receptor, each experiment is performed with a control arm consisting of an unrelated TRICEPS labelled ligand or quenched TRICEPS reagent and proteomics-based methods are used to identify biases in the labelling induced by ligand binding to its receptor.

Since most cell surface receptors are glycosylated, the TRICEPS method allows for unbiased identification of cell surface receptors following ligand binding on live cells. However, one limit of the published TRICEPS method is its reliance on the quantification of a limited subset of peptides, specifically those that are N-glycosylated. While this results in a very clean peptide mixture, it also limits the cell surface receptors that can be identified by this method, since “identifiable” cell receptors must have an N-linked glycosylation site and this site must be present in a peptide of suitable size for typical MS analysis following enzymatic digestion. Receptors that have only O-linked glycans or that contain N-linked glycans on very small or very large tryptic peptides would be difficult to identify by this method. Since the nature of the cell surface receptor is not known before these experiments, it would be ideal to design a workflow capable of identifying a wider range of proteins.

Previously, crosslinkers such as the commercially available Sulfo-SBED (Sulfosuccinimidyl-2-[6-(biotinamido)-2-(p-azidobenzamido)hexanoamido]ethyl-1,3′-dithiopropionate) have been used to identify binding partners through the transfer of a biotin moiety from a known ligand to one of its cellular binding partners^6^. Sulfo-SBED contains an amine reactive group for ligand labelling separated by a disulfide bond from a biotin and a UV-activated aryl azide crosslinking group. This configuration allows the transfer of the biotin moiety from the ligand to its crosslinked binding partner upon reduction. In practice, the Sulfo-SBED biotin transfer crosslinker has not been widely used due to technical issues associated with the UV activation, especially its low efficiency and its nonspecific crosslinking chemistry which allows for the formation of intramolecular crosslinks within the labelled ligand.

Here we have designed a heterotrifunctional crosslinker, ASB, which combines features from both TRICEPS and biotin transfer reagents. We demonstrate the ability of ASB to identify the cell surface receptors of a variety of soluble protein ligands using a biotin transfer style workflow.

## Experimental Section

### Crosslinker synthesis

All reactions were carried out at room temperature (RT) unless otherwise noted. Fmoc-cysteamine-SASRIN resin (0.4 mmol; Bachem D-2165, Lot# 1047717) was swelled in 7 mL of dimethylformamide (DMF, 15 minutes), washed further with DMF (3 × 7 mL) and deprotected with 20% piperidine in DMF (3 × 7 mL for 5–10 minutes each) before extensive washes with DMF (3 × 7 mL), dichloromethane (DCM, 1 × 7 mL), and DMF (3 × 7 mL). Coupling was accomplished through incubation of the resin with 2.5 equivalents of Fmoc-Asp(biotinyl-PEG)-OH (EMDMillipore #852113), 2.5 equivalents of HATU (1-[Bis(dimethylamino)methylene]-1H-1,2,3-triazolo[4,5-b]pyridinium 3-oxide hexafluorophosphate) and 3.5 equivalents of DIPEA (N,N-Diisopropylethylamine) in DMF for a total of 1.5 hours with an additional 1 equivalent of Fmoc reagent and HATU added after 30–45 minutes. The resin was washed extensively with DMF (3 × 7 mL). This cycle of deprotection, wash, couple, wash was repeated two additional times to couple Fmoc-NH-(PEG)2-COOH (20 atoms; EMDMillipore #851031) and Boc-aminooxyacetic acid (Boc-Aoa, Sigma #15035), respectively. For the Boc-Aoa coupling, 5 eq collidine were used to replace the DIPEA. The beads were further washed in DMF and DCM (5 × 5 mL each), dried under vacuum, and the crosslinker was cleaved off of the resin with trifluoroacetic acid (TFA)/H_2_O/triisoprolylsilane (94:4:2) supplemented with 10 equivalents of aminooxyacetic acid (Aoa, Sigma C-13408) for 2 hours^7^. The cleaved product was precipitated in cold (−20 °C) diethyl ether, resuspended in H_2_O, aliquoted and dried. The crosslinker was crudely purified by SepPak (VacRC C18-500 mg; Waters; loading = 0.025 mmol eq) and fractions were quantified using an Ellman’s assay as described^8^,^9^. Ellman’s assays estimated a yield of 47% in the crude product and 27% after purification. Aoa (10 eq) was added to the ASB containing fraction before it was aliquoted and freeze-dried. Lyophilized crosslinker was stored at −20 °C. The mass profile of the crosslinker was determined by positive electrospray mass spectrometry (MS) on a Q-TOF Ultima (Waters) following on-line desalting on nanoAcquity LC system plumbed with a C8 trap column only.

### Ligand labelling with ASB

Three separate biological repeats (BRs) were performed for each experiment. Ligands included human Integrin alpha3/CD49c Ab Monoclonal Mouse IgG1 (R&D Systems, MAB1345), human TfR (Transferrin Receptor) Ab Monoclonal Mouse IgG1 (R&D Systems, MAB2474) and human insulin (Sigma, I9278). The sdIGF1R-Fc was produced in-house. Briefly, the single-domain antibody (sdAb) was identified by panning an immunized llama library against recombinant extracellular domain of human IGF1R (Insulin-like growth factor 1 receptor) and a synthetically-produced DNA encoding this sdAb fused with an IgG1 Fc domain was expressed in CHO (Chinese hamster ovary) cells and purified by Protein A chromatography. Prior to labelling, 100 μg of ligand was buffer exchanged from its storage buffer into 50 μl of Phosphate Buffered Saline (PBS) pH 7.4 by ultrafiltration (Millipore Amicon Ultra 0.5 mL filters, 30 K (or 3K for Insulin)). For the first step, 20 nmol of a linker, LC-SPDP or PEG_4_-SPDP (Thermo Fisher), was incubated with the ligand for 30 minutes at RT. Excess N-hydroxysuccinimide groups were quenched with 20 nmol of glycine (Bio-Rad) for 30 minutes at RT and supplemented with 1 mM of ethylenediaminetetraacetic acid (EDTA). Prior to use, ASB was reduced by immobilized TCEP (tris(2-carboxyethyl)phosphine. Pierce) for >1 hour and free Aoa was removed by solid phase extraction with H_2_O and 40% acetonitrile (ACN, Empore C18 Solid phase Extraction Cartridge, 3M #4215SD). The 40% ACN fraction containing 60 μg aliquots of cleaned ASB was vacuum concentrated to remove acetonitrile before incubation with the SPDP-labelled ligand at 4 °C overnight. The ASB-labelled ligand was then buffer exchanged by ultrafiltration into PBS pH 7.4 and briefly held at 4 °C until use. Labelled ligands (~1 μg) were separated by SDS-PAGE (4–20% Mini-Protean TGX gels, Bio-Rad) under reducing conditions and visualized by Sypro Ruby (Bio-Rad) using standard procedures to ensure that there was no significant loss of the ligands during the labelling process.

### Cell culture and incubation with labelled ligands

150 mm plates of HEK293 (human embryonic kidney) or Panc-1 (human pancreatic carcinoma) cells were grown to 70-100% confluency under standard conditions in DMEM supplemented with 10% FBS and an antibiotic/antimycotic (Sigma A5955). Typically 3-4 Panc-1 plates or 5-7 HEK293 plates were used for each sample. Cells were harvested by scraping or with Accutase as suggested by the manufacturer (Sigma). Cell pellets were washed with chilled buffer (either PBS (Corning #21-040-CM) or Live Cell Imaging Solution (Life Technologies #A14291DJ) at pH 6.5 and re-suspended in chilled sodium meta-periodate (2–10 mM) in pH 6.5 buffer for 20 minutes at 4 °C. Cells were then washed 1x in chilled buffer at pH 7.4 and 1x in chilled buffer at pH 8.0. Labelled ligands were added to 6 to 10 mL of chilled buffer at pH 8.0 and incubated with the cells for 45–90 minutes at 4 °C before addition of 10 mM p-phenylenediamine (Sigma, from a 1 M dimethyl sulfoxide (DMSO) stock) followed by incubation at 4 °C for an additional 20 to 30 minutes. Cells were washed 2x in chilled buffer pH 7.4 and the pelleted cells were stored at −80 °C.

### Isolation of biotinylated proteins and trypsin digest

Cell pellets were lysed in PBS pH 7.4, 2% SDS supplemented with 1:200 (v/v) protease inhibitor cocktail (Sigma P8340) and 1:1000 (v/v) Benzonase (EMD Millipore #71205-3). Proteins were reduced with 10 mM dithiothreitol (DTT), alkylated with 37.5 mM iodoacetamide, and the reaction was quenched with an additional 10 mM DTT. Lysates were diluted 5-fold with 50 mM ammonium bicarbonate (AMBIC) and incubated with ~20 μl of UltraLink Streptavidin Resin (Pierce) for 1 hour with rotation. Streptavidin beads were washed 5x with PBS pH 7.4 + 0.5% SDS, 5x with 2 M urea in 50 mM AMBIC, and 10x with 50 mM AMBIC. Bound proteins were digested on-bead with 1 μg of trypsin (Promega V5113) in ~70 μl of 50 mM AMBIC at 37 °C overnight.

### Mass spectrometry analysis

Between 1/6^th^ to 1/10^th^ of each sample was analyzed by automated nanoLC-MS(/MS) on an LTQ-Orbitrap XL (Thermo Scientific) coupled to a NanoAcquity UPLC system (Waters). Peptides were trapped using an inline C8 precolumn (LC Packings, 161194) and C18 trap column (Waters, 186003514) and separated on a 10 cm × 100 μm I.D. C18 column (Waters, 1.7 μm BEH130C18, 186003546) at ~250 nL/minute using a 60 minute gradient (solution A: 0.1% formic acid, solution B: 100% ACN/0.1% formic acid), followed by a 9 minute equilibration at 1% solution B. Blanks with a 25 minute gradient were run between samples to minimize carryover. MS spectra were acquired in the Orbitrap between 400 and 2000 Da m/z in profile mode at 60k resolution, while data-dependent turbo CID MS/MS scans of the top 3 ions were acquired concurrently in the ion trap in centroid mode with dynamic exclusion (20s) using the following settings: isolation width = 3.0, activation Q = 0.250, activation time = 30 ms, and normalized collision energy = 35.0. All samples were injected a minimum of 2 times as technical repeats (TRs) with at least one TR using an exclude list for high abundance streptavidin peptides.

### Database searches for peptide identifications

All MS/MS spectra were assigned to peptide sequences using Mascot. Orbitrap data was converted to mzXML using Msconvert from the ProteoWizard package^10^ with the following parameters: –mzXML -32 –filter ‘peakPicking true [2,3]’. MGF files (*.mgf) were generated from the mzXML file using MzXML2Search from the Trans Proteomics Pipeline project and searched against the SwissProt database (April 2016, 550960 sequences; parameters: enzyme = trypsin; modifications = carbamidomethyl (C, fixed), oxidation (M, variable); peptide tolerance = 1.2 Da; fragment tolerance = 1.2 Da; 1 missed cleavage allowed). Peptides were subsequently filtered to remove peptides with a mascot score <35, delta mass >12 ppm, or with an oxidized methionine. As previously described^11^, we found the wide peptide mass tolerance followed by a tight mass filter led to a lower false identification rate than setting a tight mass tolerance during the search. Peptides were assigned to the protein that had the most other unique peptides identified in that BR. Only human proteins with at least 2 unique ions identified in at least 2 BRs were kept. Keratin proteins were removed.

### Quantification by label-free LC-MS

Identified peptides in each of the 3 BRs were quantified using in house software, based on MatchRx^12^. Briefly, ion current at the m/z of each identified peptide (+/−12 ppm) was extracted for 100 MS1 scans centered on the retention time of the MS/MS identification. Data for each BR was normalized based on the median intensity of peptides quantified in all MS runs. Peptide fold change was calculated based on the median intensity value from 2-4 TRs with the minimum intensity set to 50000. Protein-level p values and fold change were calculated separately for each BR by a distribution-free permutation method using the online Quantitative Proteomics p-Value Calculator (QPPC)^13^ with 2500 permutations, no multiple testing correction and no outlier removal. BR data was then combined by using the median log2 fold change between the 3 BRs and a combined p-value was calculated as described by L. Jost^14^. Proteins were identified as significantly different if they had a combined p-value equivalent to <0.05 after applying a Bonferroni correction based on the number of proteins tested. For the peptide-level volcano plots, only unique peptide sequences identified in all 3 BRs were plotted and p values for each BR were calculated by a Student’s t-test of the technical repetitions. A combined p value was calculated as described above.

## Results

### Features and synthesis of the ASB crosslinker

**Figure 1:**
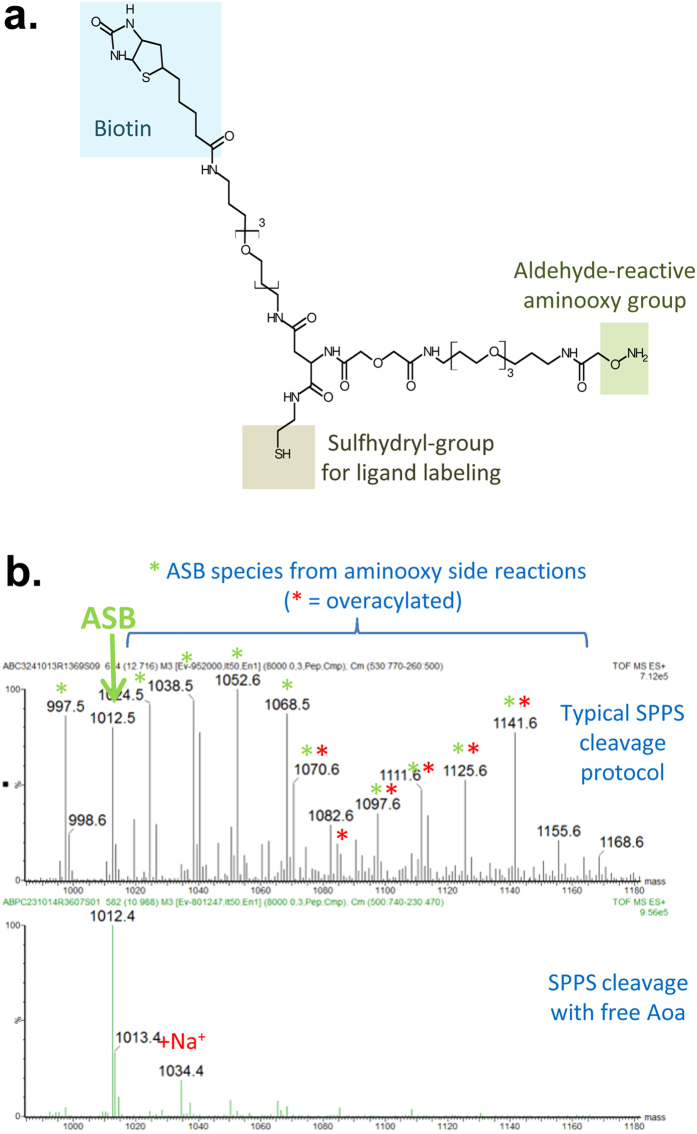
ASB crosslinker design and synthesis. (**a**) Chemical structure of ASB crosslinker highlighting its three functional groups. Both biotin and the aminooxy group are separated from the sulfhydryl by PEG-based linkers to provide separation (~33 Å) from the sulfhydryl group, which is used to link ASB to the ligand. (**b**) Mass spectrum of ASB synthesized using a typical solid-phase peptide synthesis (SPPS) protocol (top panel) and after modifications (bottom panel). The expected monoisotopic m/z for ASB is 1012.5 Da. Typical cleavage protocols resulted in masses that correspond to the addition of aldehyde contaminants to the aminooxy group of ASB (green asterisks) and to overacylation during the final coupling step leading to the addition of two aminooxy groups (red asterisks). Modifications, including addition of free aminooxyacetic acid (Aoa) during the resin cleavage step and use of collidine as the base during the final coupling reaction, greatly decreased these undesirable side products.

The chemical structure of the ASB crosslinker is shown in Fig. 1a. This crosslinker has three functional groups: (1) a sulfhydryl group for ligand labelling, (2) an aldehyde-reactive aminooxy group to allow crosslinking with oxidized glycans on the ligand receptor, (3) and a biotin moiety to allow for affinity purification. PEG-based spacer arms provide separation between the functional groups while maintaining water solubility (Fig. 1).

ASB was specifically designed for ease of synthesis using standard solid phase peptide synthesis protocols and commercially-available intermediates. During our first synthesis attempts, we found the aminooxy group to be very reactive towards contaminating aldehydes, especially under the acidic conditions required for cleavage from the solid-phase resin. Mass spectrometry analysis of the product after cleavage under typical conditions revealed several addition masses due to side reaction of the ASB aminooxy group with contaminating aldehydes (formaldehyde (+12 Da), acetaldehyde (+26 Da), acetone (+40 Da), and hydroxyacetone (+56 Da) as well as evidence of acidic hydrolysis of the amino group (-15 Da) (Fig. 1b top panel)^15^. However, in subsequent synthesis attempts, we found this over-reactivity could be minimized by adding free aminooxyacetic acid (Aoa) during resin cleavage to act as a quenching agent for contaminating aldehydes and by limiting exposure of the crosslinker to acidic conditions during purification (Fig. 1b bottom panel)^7^. In our first synthesis attempt using standard methods and Fmoc-Aoa, we also noted an additional series of undesired products that resulted from overacylation at the –O-NH_2_ group, leading to the incorporation of two Aoa residues. This can be seen in the top panel of Fig. 1b; the predicted mass of ASB with two Aoa residues is 1085.5 Da and additional peaks corresponding to this overacylated product with the addition of the aldehyde contaminants are also present. This overacylation was much improved by using a weaker base, collidine, during coupling with Boc-Aoa^16^.

### The biotin transfer method for identification of cell surface receptors

The biotin transfer method for the identification of cell surface receptors consists of three major steps which are performed in parallel for a control ligand and the ligand of interest: (1) Ligand labelling, (2) Binding-mediated crosslinking of the labelled ligand to live cells followed by solubilisation and reduction; this results in transfer of the ASB biotin moiety from the ligand to the crosslinked cellular proteins, and (3) isolation and comparative quantitation of biotinylated proteins by a proteomics-based MS method.

**Figure 2:**
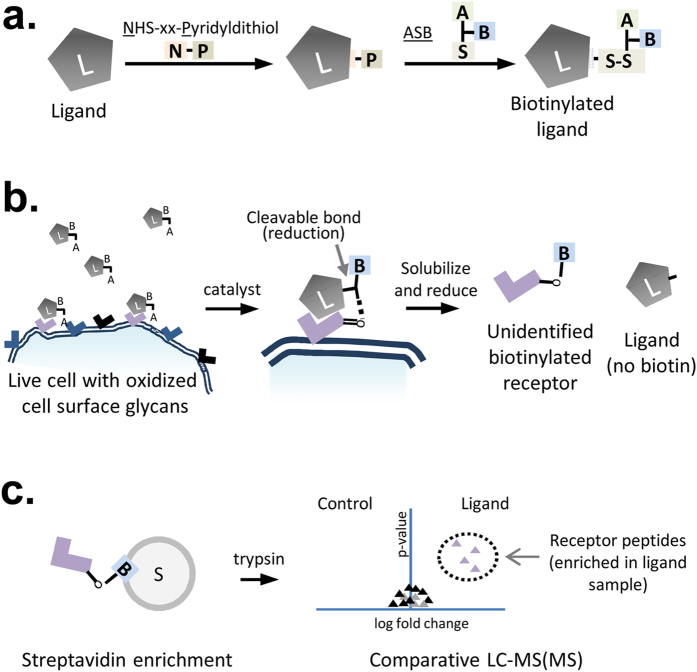
Biotin transfer workflow for identification of the ligand-binding partner. (**a)** Ligand labelling: Ligands of interest are labelled in a two-step reaction through addition of a commercial amine-to-sulfhydryl crosslinker (LC-SPDP or PEG4 version) followed by addition of ASB. Both a control ligand and a ligand-of-interest are prepared in parallel. (**b**) Biotin-transfer to cell surface receptor on periodate-treated live cells: The labelled ligand in incubated with cells to encourage ligand-directed cell surface binding prior to addition of a catalyst to promote oxime bond formation and crosslinking. Cells are then solubilized under reducing conditions resulting in the transfer of the biotin from the ligand to its crosslinked binding partner. (**c**) Isolation of biotinylated proteins and identification of enriched peptides by comparative mass spectrometry: Biotinylated proteins are immobilized on a streptavidin resin and digested with trypsin. The intensity of peptides resulting from parallel experiments with the control ligand and the ligand-of-interest are then compared using standard mass spectrometry-based proteomic workflows.

**Figure 3:**
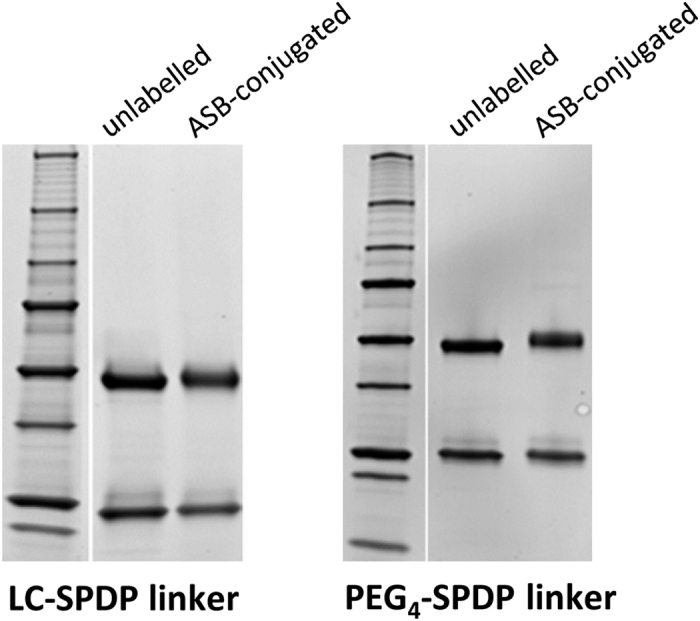
ASB labelling of a monoclonal antibody (anti-transferrin receptor) with either LC-SPDP+ASB or PEG4-SPDP+ASB leads to a visible shift in both the heavy chain and the light chain by SDS-PAGE. Total protein was visualized using Sypro Ruby under reducing conditions.

Ligand labelling is achieved through a two-step process. For the biotin transfer protocol described here, an SPDP-based amine to sulfhydryl crosslinker is used to link ASB to the ligand. The NHS ester of SPDP randomly conjugates to exposed lysine residues or the N-terminal amine on the ligand, leaving the pyridyldithiol reactive group to crosslink with the free sulfhydryl on ASB. This reaction results in a cleavable disulfide bond between the SPDP-derived linker and the ASB molecules (Fig. 2a). In these experiments, we used either LC-SPDP or PEG4-SPDP as the amine to sulfhydryl linker. The linker length provided by PEG4-SPDP is longer (25.7 Å) than LC-SPDP (15.7 Å), but both of these molecules provided comparable results for cell receptor identification using the biotin transfer method in one or more experiments with 4 different ligands (data not shown). Under the conditions used here, ligand labelling with ASB typically led to a visible upwards shift on an SDS-PAGE gel as shown for a monoclonal antibody against the transferrin receptor, where minor molecular weight shifts can be seen for both the heavy and light chain (Fig. 3).

After labelling, ASB-conjugated ligands are incubated with cells that have been pretreated with periodate in order to oxidize the exposed cell surface glycans to produce aldehydes. These cell surface aldehydes react with the aminooxy group on the ligand-bound ASB to form a covalent oxime bond with the glycosylated cell surface receptor, catalyzed by the addition of p-phenylenediamine (Fig. 2b). Next, cells are lysed under reducing conditions in the presence of SDS to solubilize all membrane proteins and cleave the ASB crosslinks, resulting in the transfer of the ASB biotin moiety from the ligand to the cell surface receptor.

In theory, binding of the ligand to a particular cell surface receptor will bias crosslinking towards that receptor; however, crosslinks can also form randomly following non-specific interactions between the cell and the ligand. Therefore, it is necessary to separate the non-specific crosslinking events from binding-mediated crosslinking events. For this reason, parallel experiments with the ligand of interest and an unrelated control ligand are compared to identify proteins that are biotinylated at a higher level in the ligand sample than in the control sample. To achieve this, biotinylated proteins from both samples are isolated by streptavidin affinity purification and digested into peptides by adding trypsin to the streptavidin beads (Fig. 2c). Proteomic-based methods can then be used to identify proteins that have different levels of biotinylation in the control and ligand sample. In this case, we quantified tryptic peptides using nano-LC-MS/MS and a label-free quantification approach based on MS signal intensity.

### Proof-of-concept for the biotin transfer workflow

To demonstrate the utility of the ASB biotin transfer workflow, we explored several ligands with known cell surface receptors, including a peptide hormone, monoclonal antibodies, and an Fc-fusion of a single-domain antibody.

**Figure 4:**
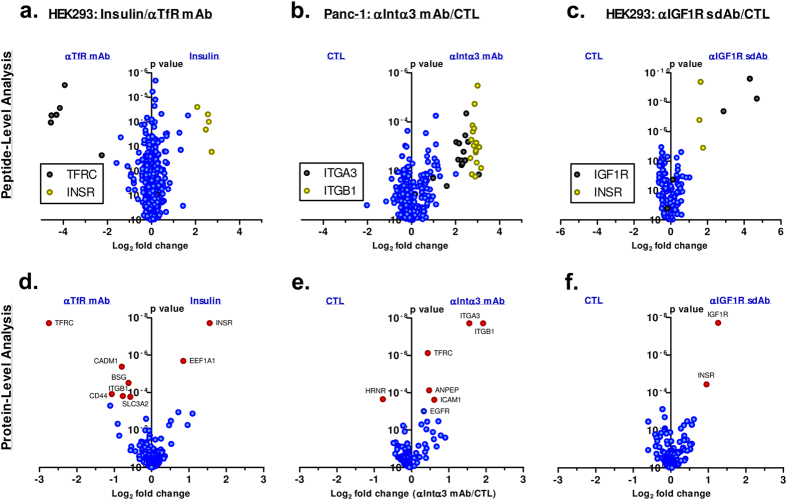
Volcano plots displaying peptide and protein-level results from three ASB biotin transfer experiments. In each case, the expected cell surface receptor of the ligand(s) of interest was identified. Ligands used include insulin (**a,d**), a monoclonal antibody against transferrin receptor (**a,d**) and against integrin alpha 3 (**b,e**), in addition to a single-domain antibody Fc fusion that was isolated by panning against recombinant IGF1R extracellular domain (**c,f**). The top panels display peptide-level results where each data point represents a unique identified peptide sequence. Data points corresponding to proteins of interest are represented in black or yellow. The bottom panels display protein-level results where each data point represents a summed result from all corresponding peptides. Proteins that were found to be significantly enriched in one sample relative to the other are highlighted in red and labelled with their HGNC symbol. Each experiment represents 3 individual biological repetitions.

In the first proof-of-concept study, we labelled the peptide hormone insulin and a commercial monoclonal antibody against transferrin receptor (anti-TfR mAb) with ASB and incubated each separately with HEK293 cells. As expected, comparison of the streptavidin-isolated proteins in these two ligand samples clearly demonstrate the enrichment of insulin receptor (INSR) and transferrin receptor (TFRC) in the insulin and anti-TfR mAb samples respectively; volcano plots of the fold-change versus p value data for this comparison is plotted at both the peptide (Fig. 4a) and protein level (Fig. 4d). Several additional proteins were also identified as significantly higher in abundance in the anti-TfR mAb sample, as represented by red dots on the volcano plot. These proteins, CADM1, BSG, ITGB1, SLC3A2, and CD44, represent “secondary” hits in the assay and may result from several different mechanisms (see Discussion).

Full quantitative and peptide identification data for all experiments can be found in Supplementary Table S1 online. In each of these experiments, many human proteins were identified which can be categorized into four distinct groups. The first group contains keratin proteins from human skin contamination during sample processing. Since keratins are often present at different levels in the two samples, peptides derived from keratin proteins have been removed prior to quantitative analysis. The second protein group contains proteins that bind non-specifically to the streptavidin beads; these are usually abundant intracellular proteins such as ribonucleoproteins, histones and other DNA-binding proteins, and cytoskeletal proteins. The third group contains naturally biotinylated proteins, such as the carboxylases. The final group contains cell surface proteins that have been biotinylated by ASB, either as background crosslinking events or in a ligand-directed manner. With the exception of the keratins, the only proteins that show consistent and significant differences between parallel experiments with two unrelated ligands are the ones that undergo ligand-directed biotinylation.

In a second proof-of-concept experiment, the ASB biotin transfer workflow was used to successfully identify the binding proteins of an anti-integrin alpha 3 monoclonal antibody (anti-Intα3 mAb) on the surface of Panc-1 cells. In this case, two proteins were identified as strongly over-represented in the sample with ASB-labelled anti-Intα3 mAb, as shown in Fig. 4b (peptide-level) and Fig. 4e (protein level) – integrin α3 (ITGA3) and integrin β1 (ITGB1). Interestingly, this antibody was originally identified following immunization with a human milk epithelial cell line. Although the antigen of this antibody has already been identified as integrin α3, this experiment shows the utility of this workflow for the identification of unknown antibody antigens resulting from cell line immunization or library panning strategies. As in the previous experiment, secondary hits were also identified, including TFRC, ANPEP, and ICAM1. In the control arm, the protein Hornerin (HRNR) was found to be more highly represented. This protein is almost certainly a contaminant as, like keratin, it is highly expressed in skin cells.

Lastly, we used the biotin transfer workflow to identify the cell surface receptor of sdIGF1R-Fc, a single-domain llama antibody fused to the Fc region of human IgG1 in HEK293 cells. This single-domain antibody binds to human IGF1R with sub-nanomolar affinity and does not cross-react with insulin receptor in surface plasmon resonance (SPR) studies (data not shown). Our results clearly identify both IGF1R and insulin receptor (INSR) as being preferentially biotinylated in the sdIGF1R-Fc sample, as can be seen in Fig. 4c and f.

## Discussion

We have demonstrated the synthesis and application of a heterotrifunctional label transfer reagent, ASB. ASB requires a 2-step labelling procedure using an amine-to-sulfhydryl linker to attach ASB to the ligand of interest. This design allows amine-based addition to the protein ligand while eliminating compatibility issues between NHS-esters and the ASB aminooxy functional group. Although this two-step labelling procedure introduces added complexity and an extra step during labelling, it also allows for greater flexibility in the use of ASB. A wide variety of commercially available linkers can be used in the first step of the labelling procedure. For example, the crosslinking distance of ASB can be modified by using longer or shorter linkers. Alternative chemistries can also be used to attach ASB to different sites on the ligand; for instance, an aldehyde-sulfhydryl reactive bifunctional crosslinker can be used to label ligands via their glycans. Lastly, ASB can be used readily in a TRICEPS-like protocol as discussed below. As with all protein conjugation methods, the addition of the ASB group may affect the ability of some ligands to bind its target. Effects on binding affinity can be elucidated by using cell-binding assays, such as flow cytometry, to compare binding of the conjugated and unconjugated ligand.

The ASB label transfer reagent is similar in many ways to the TRICEPS method presented by Frei *et al*.^2^,^3^. In addition to the biotin-transfer method described here, we have also successfully used the ASB crosslinker in a TRICEPS-like protocol by using a maleimide-based amine-sulfhydryl crosslinker (LC-SMCC) to attach ASB to the ligand via a noncleavable bond. The TRICEPS protocol results in a simpler and more specific set of peptides than the biotin transfer protocol since only peptides modified by an N-linked glycan are eluted from the streptavidin beads by PNGaseF enzyme treatment; the biotin transfer method typically provides more peptides from the putative cell receptors since proteins are solubilized using SDS and all possible tryptic peptides from bound proteins are released by the on-bead digest. However, this comes at the cost of a larger background peptide signal due to peptides from naturally biotinylated proteins, non-specifically bound proteins, and streptavidin itself. In many cases, it will be worthwhile to try both methods as they are quite complementary.

Like the TRICEPS crosslinker, ASB minimizes intramolecular crosslinking back to the soluble ligand by relying on an aldehyde reactive group to crosslink with oxidized glycans present only on the cell surface. ASB utilizes an aminooxy group for this aldehyde reactivity, while TRICEPS utilizes a protected hydrazide group. Due to the difficulty in accessing the TRICEPS reagent, we were unable to compare the efficiency of conjugation of the aminooxy group relative to the hydrazide group in TRICEPS. However, it is interesting to note that the aminooxy group has previously been shown to have higher labelling efficiency than hydrazide when labelling oxidized glycans^17^.

In the proof-of-concept studies described here, we successfully identified the expected cell surface receptor using ASB as a biotin transfer reagent. Interestingly, in several instances we also identified “secondary” hits – cell surface receptors that appeared to be moderately overrepresented in the ligand relative to the control. There are a few mechanisms that might explain these secondary hits, including (1) co-localization with the primary receptor, (2) off-target or non-specific binding of the ligand, and/or (3) changes in cell surface expression levels induced by the ligand or control molecule. In many cases, we expect that the secondary hits represent near-neighbors of the ligand receptor that are preferentially crosslinked due to their close proximity to the receptor-bound ligand. This is especially clear when the expected cell surface receptor forms a heterodimer. In this situation, we typically see an equally strong biotin transfer to both heterodimer partners, as can be seen with the α3/β1 integrin (ITGA3/ITGB1) heterodimer that is recognized by the anti-intα3 mAb. We see a similar phenomenon with the single-domain Fc fusion protein that recognizes IGF1R where we see a strong biotin transfer to both IGF1R and the insulin receptor (INSR). IGF1R and INSR are known to form heterodimers^18^,^19^. While it is also possible that the IGF1R single-domain antibody may bind to insulin receptor directly in an off-target effect (58% of the protein sequence is identical between human IGF1R and insulin receptor), this is unlikely in this case since sdIGF1R-Fc does not show any crossreactivity to insulin receptor by SPR. It is interesting to note that using insulin as a ligand did not appear to mediate biotin transfer to IGF1R in these same cells. This is consistent with the finding that insulin binds with low affinity to IGF1R/insulin receptor heterodimers^20^.

The near-neighbor effect is also evident beyond the context of heterodimer formation. For instance, transferrin receptor has been shown to interact with α3/β1 integrin heterodimers, suggesting that integrin beta1 (ITGB1) is preferentially biotinylated by the ASB-labelled anti-TfR mAb due to the close proximity of transferrin receptor and α3/β1 integrin in the cell membrane^21^. Reciprocally, when an anti-α3/β1 integrin monoclonal was labelled with ASB, it was shown to preferentially biotinylate the transferrin receptor (TFRC). Lending extra weight to the near-neighbor hypothesis, a couple of other known interactors of α3/β1 integrin were also found as secondary hits in this experiment, including EGFR^22^,^23^ and, to a lesser extent, basigin (BSG)^24^. In the future, the biotin transfer workflow may prove useful to identify interacting proteins within cell membranes, an area that is technically difficult using traditional protein-protein interaction workflows, such as co-immunoprecipitation.

In addition to near-neighbor and off-target effects, the secondary hits could also represent a signaling-induced change in cell surface localization, resulting in differences in non-specific crosslink formation. For instance, insulin is well-known to affect cell surface expression levels of several proteins through modulation of internalization^25^,^26^,^27^ or ectodomain shedding^28^,^29^. For this reason, we speculate that some of the secondary hits seen in Fig. 4a in the ASB-labelled transferrin receptor antibody sample may result from decreased cell surface expression induced by insulin signaling. Similarly, increased biotinylation of EEF1A1, an elongation factor, may result from its translocation to the cell surface during anoikis^30^. Although all incubations were performed at 4 °C, the level of insulin exposed to the cells during binding is very high (approximately 15 μM) and even brief exposure to room temperature may allow for some cell response. Effects due to ligand signaling could be minimized by using unrelated control molecules in each biological repetition to prevent these types of expression changes from leading to a statistically significant result.

## Conclusions

In conclusion, we have introduced a new biotin transfer agent and demonstrated its ability to identify the cell surface receptors of several types of ligands, including a peptide hormone, monoclonal antibodies, and single-domain antibodies. In the future, it would be interesting to combine the strengths of the previously described TRICEPS method and the biotin transfer method described here. This could be accomplished by adding an orthogonal cleavage site, such as a periodate cleavable site^31^, to the ASB molecule that would allow for specific elution of crosslinked biotinylated receptor proteins off of the streptavidin beads prior to trypsin digest.

## Additional Information

**How to cite this article**: Tremblay, T.-L. and Hill, J. J. Biotin-transfer from a trifunctional crosslinker for identification of cell surface receptors of soluble protein ligands. *Sci. Rep.* **7**, 46574; doi: 10.1038/srep46574 (2017).

**Publisher's note:** Springer Nature remains neutral with regard to jurisdictional claims in published maps and institutional affiliations.

## Supplementary Material

Supplementary Table S1
